# Chronic traumatic encephalopathy in a neuropathologically confirmed Creutzfeldt–Jakob disease cohort

**DOI:** 10.1111/bpa.70156

**Published:** 2026-10-14

**Authors:** Lluc Bertran, Iban Aldecoa, Arlène Sindikubwabo, Núria Vidal, Rafael Blesa, Mercè Martínez‐Corral, Carlos Nos, Verónica Santiago, Andrea González, Álex Iranzo, Christopher J. Nowinski, Ann C. McKee, Raquel Sánchez Valle, Laura Molina‐Porcel

**Affiliations:** ^1^ Faculty of Medicine and Health Science Hospital Clínic, Universitat de Barcelona Barcelona Spain; ^2^ Department of Pathology, Biomedical Diagnostic Center (CDB) Hospital Clínic, Universitat de Barcelona Barcelona Spain; ^3^ Neurological Tissue Bank Biobank‐Hospital Clínic‐FRCB‐IDIBAPS Barcelona Spain; ^4^ Department of Health Care HOGENT University of Applied Sciences and Arts Ghent Belgium; ^5^ Sant Pau Memory Unit Hospital de la Santa Creu i Sant Pau – Biomedical Research Institute Sant Pau Barcelona Spain; ^6^ Neurology Department HM Sant Jordi Hospital Barcelona Spain; ^7^ Department of Public Health in Catalonia General Subdirectorate of Surveillance and Response to Emergencies in Public Health Barcelona Spain; ^8^ Sleep Unit, Neurology Service, FRCB‐IDIBAPS, CIBERNED CB06/05/0018‐ISCIII Hospital Clínic, Universitat de Barcelona Barcelona Spain; ^9^ Boston University Alzheimer's Disease Research Center and Boston University CTE Center, Boston University Chobanian & Avedisian School of Medicine Boston Massachusetts USA; ^10^ Concussion & CTE Foundation Boston Massachusetts USA; ^11^ Alzheimer's Disease and Other Cognitive Disorders Unit, Neurology Service, FRCB‐IDIBAPS, CIBERNED CB24/05/0004 ISCIII Hospital Clínic, Universitat de Barcelona, Institut de Neurociències Barcelona Spain

**Keywords:** chronic traumatic encephalopathy, contact sports, Creutzfeldt–Jakob disease, neuropathology, phosphorylated tau, postmortem brain analysis, prion diseases

## Abstract

Chronic traumatic encephalopathy (CTE) is a tauopathy associated with repetitive head impacts, experienced most commonly in contact sports; however, its relationship with prion diseases remains unclear. Postmortem CTE screening using phosphorylated tau staining was conducted at the Neurological Tissue Bank of Hospital Clínic‐IDIBAPS on hippocampal and frontal cortex samples from patients with neuropathologically confirmed Creutzfeldt–Jakob disease (CJD), blinded to patients' clinical histories. In positive and suggestive cases, the analysis was expanded in accordance with the NINDS/NIBIB consensus criteria for CTE, and clinical and neuropathological information was reviewed. CTE was detected in 3 out of 212 CJD cases (1.4%), all with severe CTE pathology: 2 former soccer players (one of them an elite player) and 1 former rugby player. All had started playing sports between the first and second decades of life and ended in the fifth. The clinical presentation of CJD was heterogeneous, with different initial symptoms in each case. Neuropathological examination revealed an MM1/MV1 histotype in the soccer players and a VV2 histotype in the rugby player, the first‐ and second‐most common variants in CJD, respectively. These three cases illustrate the coexistence of CTE and CJD across heterogeneous clinicopathological presentations. Further studies are needed to determine whether shared mechanisms may contribute to the coexistence of both pathologies.

## Graphical abstract

Systematic neuropathological screening of 212 patients with Creutzfeldt–Jakob disease (55% women) identified chronic traumatic encephalopathy in three cases (1.4%), all men with a history of contact sports. These findings represent the first neuropathologically confirmed chronic traumatic encephalopathy cases reported in Spain.

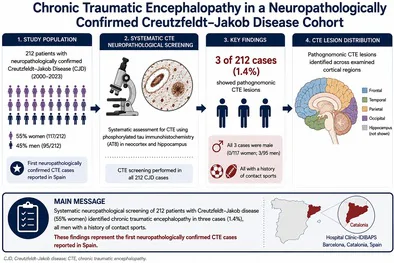

## 1 INTRODUCTION

Contact sports are associated with an increased risk of neurodegenerative diseases [1, 2, 3]. Among these, chronic traumatic encephalopathy (CTE) has emerged as the condition most consistently linked to repetitive head impacts [4].

First described in 1928 in boxers as “punch‐drunk syndrome,” later termed “pugilistic dementia” [5, 6], CTE is currently recognized neuropathologically as a tauopathy characterized by the perivascular accumulation of hyperphosphorylated tau in neurons at the depths of cortical sulci [7, 8]. According to the revised National Institute of Neurological Disorders and Stroke (NINDS) and National Institute of Biomedical Imaging and Bioengineering (NIBIB) consensus criteria (2021), a single pathognomonic lesion is sufficient for diagnosis, and disease severity is staged by the distribution of the p‐tau pathology [8].

The prevalence of CTE in the general population is unknown. In autopsy studies of contact sport athletes who donated their brains to brain banks, the prevalence is high, ranging from 54% to 99% [9, 10, 11]. In contrast, reported frequencies range from 0% in a European community‐based aging cohort to 2.2% in a routine neuropathology series [12, 13].

CTE often coexists with other neurodegenerative conditions, such as Alzheimer's disease, Lewy body pathology, or amyotrophic lateral sclerosis [8, 14]. This overlap complicates clinicopathological correlations and highlights the importance of exploring comorbidities.

Creutzfeldt–Jakob disease (CJD) is a rapidly progressive and fatal neurodegenerative disorder characterized neuropathologically by spongiform changes and extracellular deposition of prion protein in the brain. The most common histotypes are MM1 and VV2, corresponding to patients homozygous for methionine (MM1) or valine (VV2) at codon 129 of the *PRNP* gene, each associated with distinct molecular, neuropathological, and clinical features [15].

Despite the growing literature on CTE and its comorbidities, its relationship with prion diseases remains largely unexplored. To date, only one previous study reported three cases of concomitant CTE and CJD [16].

In this study, we investigated the frequency of CTE in a series of patients with neuropathologically confirmed CJD and to explore the clinical and neuropathological characteristics of CTE‐positive cases.

## 2 MATERIALS AND METHODS

### 2.1 Study design

A retrospective cross‐sectional study was conducted in patients with neuropathologically confirmed CJD available up to January 1, 2024, at NTB‐Hospital Clínic‐IDIBAPS. Brain donation was systematically proposed to all patients with suspected rapidly progressive dementia of possible prion origin. Genetic forms were excluded. In addition, atypical cases with prolonged disease duration, in which CJD had not been initially suspected but were subsequently confirmed neuropathologically, were also incorporated.

CTE screening was performed blinded to the neuropathological diagnosis and clinical information, following a reduced version of the second NINDS/NIBIB consensus criteria [7]. Immunohistochemical staining for phosphorylated tau (p‐tau; AT8, Thermo Fisher Scientific, USA) was evaluated in the hippocampus and frontal cortex of all subjects in the brain bank cohort.

The assessment focused on the pathognomonic lesion of CTE, defined as perivascular aggregates of hyperphosphorylated tau in neurons, with or without thorn‐shaped astrocytes, located at the depth of a cortical sulcus and not restricted to the subpial or superficial regions [7, 8].

In cases showing suggestive features (e.g., tau pathology at the sulcal depths or superficial cortical neurofibrillary tangles without amyloid‐β), the analysis was expanded to include additional brain regions recommended by the consensus criteria: middle frontal gyrus, superior and middle temporal gyri, inferior parietal lobule, hippocampus, amygdala, basal ganglia, thalamus, midbrain (including substantia nigra), pons (including locus coeruleus), medulla oblongata, and cerebellar cortex when paraffin‐embedded tissue blocks were available [7]. Additional cortical regions, including the occipital cortex, were also examined. In cases fulfilling diagnostic criteria for CTE, CTE severity was determined according to the second NINDS/NIBIB consensus criteria, and the extent of pathognomonic CTE pathology was additionally assessed using the McKee staging system [17]. The assessment included additional p‐tau staining in the amygdala, thalamus, mammillary bodies, and dentate nucleus of the cerebellum [7]. The presence of pathognomonic CTE lesions was recorded for each examined region, together with the number of sections examined.

### 2.2 Clinicopathological characterization

The clinical and neuropathologic data from all cases with positive CTE screening were reviewed. Clinical variables included sex, age at disease onset, age at death, history of repetitive head injury, exposure to contact sports and duration of practice, and clinical presentation.

Neuropathological characterization was performed according to the standard protocols of the Neurological Tissue Bank [18], and classifications were updated when necessary according to current diagnostic criteria. The assessment included the presence and subtype of CJD [15, 19], the ABC score for AD neuropathologic change according to National Institute on Aging–Alzheimer’s Association (NIA‐AA)/NINDS criteria [20], Braak staging of tau pathology (I–VI) [21], Thal phase for Aβ amyloid deposition (0–5) [22], and Consortium to Establish a Registry for Alzheimer's Disease (CERAD) score for neuritic plaques (0–3) [23]. α‐Synuclein pathology was evaluated according to the McKeith criteria [24], and TDP‐43 pathology was assessed with Limbic‐predominant age‐related TAR DNA‐binding protein 43 (TDP‐43) encephalopathy neuropathological change (LATE‐NC) classification [25].

## 3 RESULTS

### 3.1 Screening and frequency of CTE in CJD cases

The sample consisted of 212 individuals diagnosed with CJD, of whom 55% (*n* = 117) were women. Screening of the frontal cortex and hippocampus identified three men with pathognomonic CTE lesions. Eight cases with suggestive features were ruled out for CTE with expanded sampling [7]. The three confirmed cases exhibited high‐grade CTE pathology. From this point forward, the confirmed cases will be referred to as Case 1, Case 2, and Case 3. A summary of the clinical and neuropathological characteristics of these cases, together with previously reported cases, is provided in Tables 1 and 2.

**TABLE 1:** Clinical characteristics of reported cases with coexisting CJD and CTE.

Feature	Case 1	Case 2	Case 3	Case 4^a^	Case 5^a^	Case 6^a^
Gender	Male	Male	Male	Male	Male	Male
Age at disease onset	Late 60s	Mid 60s	Early 80s	79 years	64 years	48 years
Age at death	Early 70s	Mid 60s	Early 80s	84 years	68 years	48 years
Primary source of RHI exposure	Soccer	Soccer and futsal	Rugby	Football and military service	Football	Multiple head traumas, fistfights, and motor vehicle accidents
Sports history and duration of exposure	Soccer from childhood for >50 years, including >20 years at elite level	Soccer from childhood to his 30s, followed by futsal as goalkeeper until the mid‐40s; >30 years of practice	Rugby from late adolescence to mid‐adulthood; 28 years of practice, including international‐level competition	Football from high school to NFL; a total of 9 years of practice	Football from high school to NFL; a total of 18 years of practice	Not a professional athlete; multiple TBI exposures
Symptoms	Progressive cognitive decline (6 years), spatiotemporal disorientation, seizures, myoclonus, akinetic mutism (3 months)	Right upper limb numbness, tremor, alien hand phenomenon, aphasia (3 months)	Gait instability, disorientation, visual hallucinations, myoclonus (3 months)	Anger, memory impairment, motor symptoms, akinetic mutism	Forgetfulness, aphasia, paranoia, gait disturbance, rapid cognitive decline	Ophthalmoplegia, confusion, rigidity, hallucinations, rapid neurological deterioration

*Note*: Cases 1–3 correspond to the present study.

Abbreviations: CJD, Creutzfeldt–Jakob disease; CTE, chronic traumatic encephalopathy; NFL, National Football League; RHI, repetitive head impacts; TBI, traumatic brain injury.

^a^ Cases 4–6 were previously reported by Nemani et al. [16].

**TABLE 2:** Neuropathological features of patients with coexisting CJD and CTE.

Feature	Case 1	Case 2	Case 3	Case 4^a^	Case 5^a^	Case 6^a^
Age at death	Early 70s	Mid 60s	Early 80s	84 years	68 years	48 years
Brain weight (unfixed)	1200 g	1635 g	1210 g	Not reported	Not reported	Not reported
Macroscopic pathology	Diffuse atrophy, especially in the hippocampus	Mild generalized edema; no significant atrophy	Mild–moderate frontoparietal atrophy; minimal in amygdala, hippocampus and cerebellum	Cortical and cerebellar atrophy	Widespread severe cortical atrophy	Most severe atrophy in occipital and cerebellum than in the frontal, parietal and temporal cortices
PrP immunohistochemistry	Synaptic pattern, predominantly affecting cerebellar and occipital cortices	Diffuse synaptic pattern, most intense in occipital and cerebellar cortex with a “patchy perivacuolar” pattern	Widespread synaptic and perineuronal PrP deposits, mainly in cerebellum.	Synaptic pattern in cerebellar cortex, and coarse pattern in temporal cortex	Coarse fine granular pattern, with mature plaques and plaque‐like formations	Synaptic pattern in neocortical regions with “brush‐stroke” cerebellar pattern
McKee CTE stage	III	I	III	IV	II	II
Bieniek CTE severity	High	High	High	High	Low	Low
Braak stage	III	II	III	III–IV	I–II	I–II
Pathognomonic CTE lesions	2/2 frontal	1/2 temporal + 1/1 fusiform	2/4 frontal +1/2 temporal	Motor, temporal, and parietal cortices	Occasional perivascular clusters at sulcal depths; region not specified	Frontal, parietal, and temporal cortices
ABC score	A3B2C2	A1B1C0	A1B2C2	A2B2C0	A2B1C0	A1B1C0
Final neuropathological diagnosis	sCJD (MM/MV1) + high‐grade CTE + intermediate AD‐NC + LATE	sCJD (MM/MV1‐MM2C) + high‐grade CTE	sCJD (VV2) + high‐grade CTE + intermediate AD‐NC	sCJD (MV1‐2C) + high‐grade CTE + intermediate AD‐NC + LATE + α‐syn (olfactory bulbs)	sCJD (MV2K‐C) + low‐grade CTE	sCJD (MM1/MV1) + low‐grade CTE

*Note*: Cases 1–3 correspond to the present study. For Cases 1–3, pathognomonic CTE lesions are reported as the number of sections showing the lesion/number of sections examined for each anatomical region. At least one section from the parietal and occipital cortices, two sections from the frontal cortex, and at least one section from the temporal cortex and hippocampal/fusiform region were examined for the presence of pathognomonic CTE lesions. For Cases 4–6, the anatomical distribution of pathognomonic CTE lesions is reported as described by Nemani et al. [16], as the number of sections examined for each region was not reported. Bieniek CTE severity for Cases 4–6 was retrospectively assigned by the authors based on the reported McKee CTE stage.

Abbreviations: ABC score (A: amyloid, B: Braak, C: CERAD); AD‐NC, Alzheimer disease neuropathologic changes; CJD, Creuzfeld–Jakob disease; CTE, chronic traumatic encephalopathy; LATE, limbic‐predominant age‐related TDP‐43 encephalopathy; PrP, prion protein; sCJD, sproadic Creuzfeld–Jakob disease.

^a^ Cases 4–6 were previously reported by Nemani et al. (2018) [16].

### 3.2 Clinical and neuropathological features of CJD cases with CTE

#### 3.2.1 Case 1

A man in his late 60s presented with progressive cognitive impairment initially characterized by memory loss, later evolving to include disorientation and language deficits. He was a former professional soccer player who had over five decades of soccer participation beginning in childhood, including more than 20 years of elite‐level competition as a striker.

Neuropsychological assessment revealed deficits in memory, language, orientation, and attention. Brain MRI showed severe hippocampal atrophy.

Six years after symptom onset, the patient experienced rapid neurological deterioration, including gait instability, myoclonus, and akinetic mutism. EEG showed periodic epileptiform discharges, and cerebrospinal fluid 14–3–3 was positive. He died 3 months later with a clinical diagnosis of possible Alzheimer's disease and CJD.

Neuropathological examination revealed a brain weighing 1235 g, with diffuse brain atrophy, predominantly affecting the hippocampus. Histological analysis demonstrated coexistent prion disease (sporadic CJD, MM/MV1 subtype) and extensive tauopathy consistent with high‐stage CTE.

Prion pathology was characterized by spongiform change, neuronal loss, and gliosis, with a predominantly synaptic PrP immunostaining pattern affecting mainly cerebellar and occipital cortices.

AT8 immunostaining revealed extensive tauopathy with predominant astroglial involvement and moderate neurofibrillary pathology, affecting mainly the frontal cortex, limbic regions, basal ganglia, and rostral brainstem. Neocortical findings included granular fuzzy astrocytes, thorn‐shaped astrocytes, as well as perivascular neuronal tau aggregates at the depths of cortical sulci, consistent with the pathognomonic lesion of CTE. Pathognomonic CTE lesions were identified in 2 of 2 frontal cortex sections examined (Table 2). Tau pathology extended to the hippocampus, amygdala, thalamus, hypothalamus, substantia nigra, and brainstem, and included both 3R and 4R isoforms, with a relative predominance of 4R in astroglial pathology. Other tau pathology included p‐tau‐positive astrocytes, coiled bodies, and globular glial inclusions in frontal and temporal white matter characteristic of aging‐related tau astrogliopathy.

Additional findings included intermediate AD neuropathologic changes (A3B2C2), mild cerebral amyloid angiopathy, and limbic TDP‐43 pathology (LATE‐NC Stage 3). α‐Synuclein pathology was absent.

#### 3.2.2 Case 2

A man in his 60s, with hypertension, Type 2 diabetes mellitus, and a history of amateur soccer playing from childhood to his mid‐40s, with participation in futsal as a goalkeeper from his 30s onward, presented with a 2‐month history of progressive neurological symptoms, initially sensory disturbances followed by right upper limb myoclonus and dysmetria.

Brain MRI revealed mild diffusion abnormalities in the left parietal, insular, and frontal cortices. EEG showed periodic lateralized epileptiform discharges in the left hemisphere. Cerebrospinal fluid (CSF) analysis was negative for 14–3–3 protein.

The disease course was rapidly progressive. Within 1 month, he developed alien hand phenomenon, asymmetric motor impairment (right‐predominant), expressive aphasia, and impaired gaze, progressing to somnolence and minimal verbal output. The final antemortem clinical diagnosis was CJD.

On gross examination, the brain weighed 1635 g and exhibited mild generalized edema with discrete vermian cerebellar atrophy.

Histopathological examination demonstrated coexistent prion disease (sporadic CJD, MM/MV1 subtype with M2C foci) and high‐grade CTE. There was widespread neuronal loss, astrogliosis, and microglial activation, with mild‐to‐moderate cortical spongiform change characterized by small‐ to medium‐sized vacuoles across cortical layers, most prominent in the occipital cortex, along with isolated foci of large confluent vacuoles supporting the presence of M2C features. The basal ganglia and cerebellum were also involved, with prominent spongiform changes in the cerebellar molecular layer. Brainstem nuclei were largely spared.

PrP immunohistochemistry showed diffuse synaptic deposits, most intense in the occipital cortex and cerebellum, with focal perivacuolar staining. Western blot confirmed PrP Type 1.

AT8 immunostaining revealed predominantly astroglial tau pathology with associated neurofibrillary changes, including neurofibrillary tangles and neuropil threads. Perivascular neuronal tau aggregates at the depths of cortical sulci were identified in the inferior temporal cortex, consistent with the pathognomonic lesion of CTE. Pathognomonic CTE lesions were identified in one of two temporal cortex sections and one of one fusiform gyrus sections examined. Additional tau pathology involved hippocampal and parahippocampal regions, with minimal involvement of the basal nucleus of Meynert and neurons of the locus coeruleus. Findings were diagnostic of high‐stage CTE.

Low AD neuropathologic changes were present (A1B1C0). No TDP‐43 or α‐synuclein pathology was detected.

#### 3.2.3 Case 3

A man in his early 80s, a former elite rugby player active from his late teens to mid‐40s, presented with gait instability 3 weeks after lumbar surgery, followed by rapid neurological deterioration over 2 months. His medical history included dyslipidemia, cardiac arrhythmia, hypertrophic cardiomyopathy, and lumbar spinal stenosis.

On admission, he was somnolent and partially oriented, with dyscalculia, impaired praxis, hypofluent speech, gait impairment, and retropulsion. Brain MRI showed diffusion hyperintensity in the right caudate nucleus, T2/FLAIR periventricular white matter hyperintensities, moderate cortico‐subcortical atrophy, and left cerebellar atrophy. CSF was negative for infection, onconeural antibodies, and 14–3–3 protein, and EEG showed diffuse slowing.

During hospitalization, he deteriorated rapidly over 72 h, developing quadriplegia, incoherent speech, visual hallucinations, lingual myoclonus, and paralytic ileus. He died 3 months after symptom onset.

On gross examination, the brain weighed 1210 g and showed mild–moderate frontoparietal atrophy, with minimal involvement of the amygdala, hippocampus, and cerebellum. Histology revealed coexistent prion disease (sporadic CJD, VV2 subtype) and high‐grade CTE. Spongiform encephalopathy predominantly affected subcortical and cerebellar regions, with moderate‐to‐severe involvement of the cerebellar cortex, hippocampal CA1, subiculum, amygdala, thalamus, basal ganglia, and brainstem, while neocortical changes were mild. Spongiform changes were characterized by predominantly small vacuoles with occasional medium‐sized forms. Marked neuronal loss and gliosis consistent with hippocampal sclerosis were present.

Immunohistochemistry showed widespread synaptic and perineuronal PrP deposits, predominantly affecting subcortical and cerebellar regions, consistent with the VV2 subtype.

Tau pathology showed neuronal and glial involvement, including perivascular neuronal tau aggregates at the depths of frontal, temporal, and insular sulci, consistent with the pathognomonic lesion of CTE. Pathognomonic CTE lesions were identified in two of four frontal cortex sections and one of two temporal cortex sections examined. The astroglial component was characterized by thorn‐shaped and granular fuzzy astrocytes in affected neocortical regions. Mild superficial neurofibrillary pathology was observed in the lateral temporal cortex. Additional involvement of mammillary bodies, thalamus, and parietal cortex was present, with occasional neurofibrillary tangles in the substantia nigra, midbrain tegmentum, and basal nucleus of Meynert, and less frequent involvement of the putamen and dentate nucleus, diagnostic for high‐stage CTE. Moderate AD neuropathologic changes were present (A1B2C2), along with limbic TDP‐43 pathology (LATE‐NC stage 2). Occasional α‐synuclein‐positive neurites were observed in the basal nucleus of Meynert.

Representative neuropathological findings, including spongiform changes, PrP deposition, and CTE‐related tau pathology, are shown in Figure 1.

**FIGURE 1:**
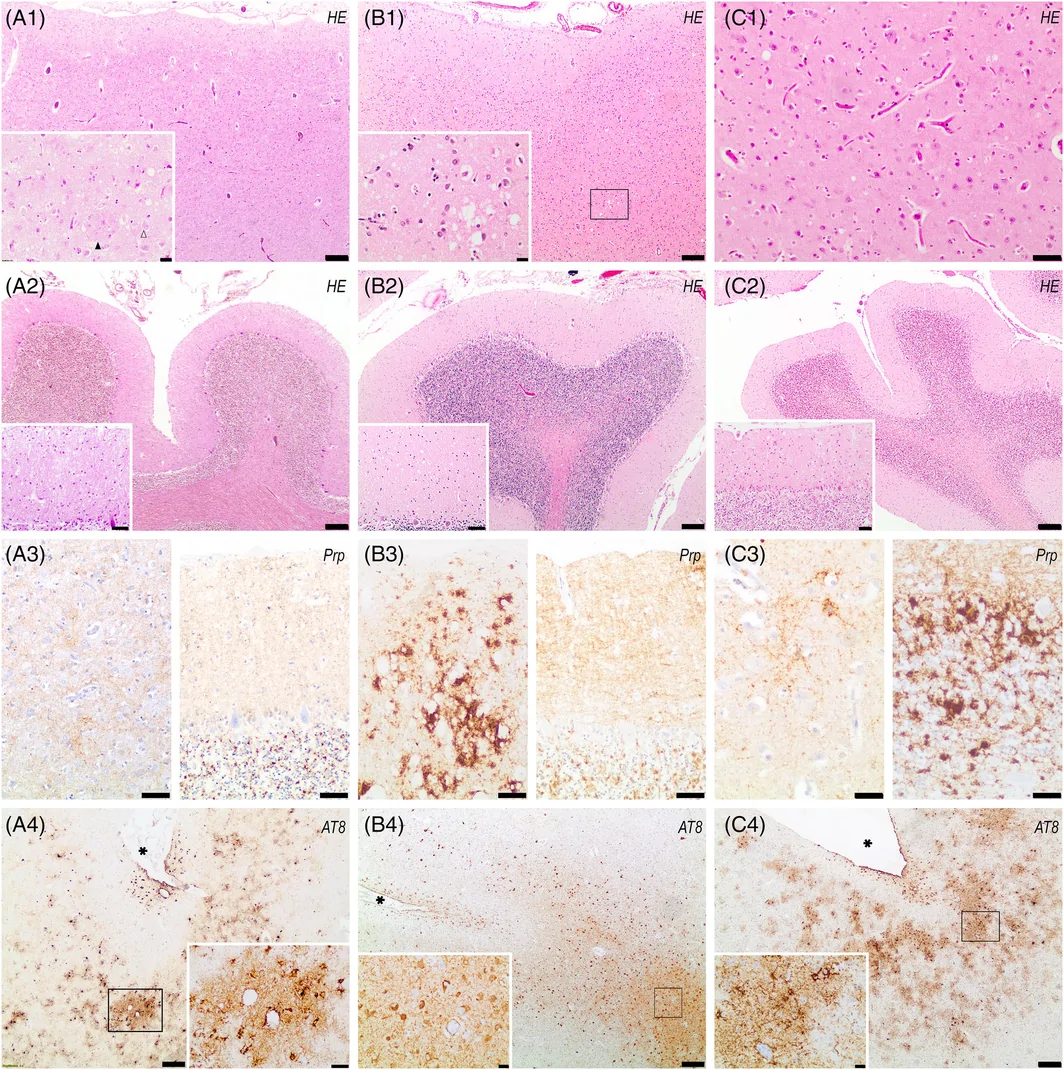
Neuropathological features in prion disease and CTE across three cases. Each column represents one case. Row 1 (A1–C1): Hematoxylin and eosin (H&E) staining of the frontal cortex. Cases A and B (MM/MV1 histotype) show moderate gliosis, neuronal loss, and spongiform change with small vacuoles. Cases A also displays amyloid plaques (black arrowhead) and neurofibrillary tangles (white arrowhead, inset). Case B shows a focal area with larger confluent vacuoles suggestive of MM2C involvement (inset). Case C (VV2 histotype) shows mild gliosis and smaller vacuoles. Row 2 (A2–C2): H&E staining of the cerebellum. All cases show degeneration with non‐confluent vacuoles in the molecular layer (inset), gliosis, and Purkinje cell loss, particularly marked in Case C. Row 3 (A3–C3): PrP immunohistochemistry. Cases A and B show diffuse “synaptic” PrP deposition in the cortex and cerebellum. Case B includes perivacuolar staining in MM2C‐like areas. Case C shows mild perineuronal PrP positivity in the frontal cortex and dense plaque‐like PrP deposits in the cerebellar granular layer. Row 4 (A4–C4): Pathognomonic CTE lesions in cortical sulci (asterisks) from frontal (A4, C4) and inferior temporal cortex (B4), showing perivascular neuronal and astroglial phosphorylated tau aggregates (insets). Scale bars: 200 μm (A1, B1, A2–C2, A4–C4); 50 μm (C1, A3–C3, insets A2–C2); 20 μm (insets A1, B1, A4–C4).

Across the three cases, pathognomonic CTE lesions were identified in multiple cortical regions, although their distribution and extent varied between cases (Table 2). McKee staging was III, I, and III for Cases 1–3, respectively, whereas all three cases were classified as high severity according to the Bieniek criteria.

## 4 DISCUSSION

To the best of our knowledge, this is the first systematic screening for CTE in neuropathologically confirmed CJD cases. These cases also represent the first neuropathologically confirmed diagnoses of CTE reported in Spain. Prior to this work, only one Spanish study had described four clinically suggestive CTE cases based on a history of traumatic brain injury (TBI), but none were neuropathologically confirmed [26].

We screened 212 subjects with neuropathologically confirmed CJD and identified three cases with high‐stage CTE, representing a frequency of 1.4%. A previous study reported three cases with coexisting CTE and CJD, including two identified within the UNITE Brain Bank (2/55, 3.6%) and one incidentally found at the National Prion Disease Pathology Surveillance Center [16]. Although the Boston University CTE Center has substantially expanded since the publication of Nemani et al., no additional cases of concomitant CJD have been identified despite routine PrP immunohistochemical screening. Patients with known or suspected prion disease are generally referred to the National Prion Disease Pathology Surveillance Center, which may limit the opportunity to identify CJD within the BU CTE cohort. Our findings provide the first estimate of CTE occurrence derived from systematic screening in a defined CJD cohort.

The interpretation of this frequency presents inherent limitations. There is no reference prevalence of CTE in the general Spanish population, and available population‐based studies derive from regions with different exposure patterns to contact sports, limiting direct comparisons. Reported prevalences in unselected cohorts range from 0% in a European community‐based aging cohort to approximately 0.6–0.8% in community‐based and autopsy‐based cohorts from the United States, Brazil, and Australia, supporting the notion that incidental CTE is rare in community populations [13, 27, 28, 29]. In this context, our observed frequency is higher; however, this difference should be interpreted with caution given differences in study design and population characteristics.

The clinical presentation of CJD was heterogeneous among our three cases, with different initial symptoms in each patient. Neuropathological examination revealed MM1/MV1 histotype in the soccer players and VV2 histotype in the rugby player, the two most common sporadic CJD variants. This heterogeneity is consistent with Nemani et al., who reported that disease‐related prion protein properties and histopathological phenotype in CTE + CJD cases did not differ from CJD‐only controls, suggesting that CTE and CJD may coexist as independent pathological processes [16]. A detailed comparison of clinical characteristics and neuropathological findings across all reported cases, including those three from the present series and previously published cases, is provided in Tables 1 and 2.

CTE has been widely reported in athletes from various contact sports. Our cases include the first reported diagnoses of prion disease in soccer and rugby players, and notably, the first documented case of CTE in a futsal player. Contrary to the common assumption that futsal involves fewer head impacts due to its smaller field and indoor setting, recent evidence demonstrates that the incidence rate of headers in futsal is comparable to that of traditional soccer (IR 32.9 vs. 36.9 per 1000 match hours) [30]. Moreover, the rate of potential head injuries requiring medical attention is actually higher in futsal (122.3 vs. 76.4 per 1000 match hours), with unintentional ball‐to‐head impacts being proportionally more frequent (16.7% vs. 6%) [31]. The affected individual played both futsal and traditional soccer throughout his career, increasing cumulative repetitive head impacts (RHI) exposure. These findings suggest that futsal players may have comparable or even greater RHI exposure than traditional soccer players, warranting their inclusion in CTE surveillance programs.

A fundamental question arising from our findings is whether repetitive trauma could act as a risk factor for the development of sporadic CJD. Early epidemiological studies suggested an association between physical trauma and CJD [32]. However, these observations have not been confirmed in more rigorous contemporary investigations, and they likely reflected unrecognized iatrogenic transmission rather than trauma‐related mechanisms [33]. Notably, the vast majority of CJD cases in our cohort showed no evidence of CTE.

The interpretation of tau pathology in the setting of CJD also requires caution. Kovacs et al. reported a broad spectrum of tau pathology in sporadic CJD, including neuronal and glial tau pathology compatible with primary age‐related tauopathy and ageing‐related tau astrogliopathy [34]. Therefore, the presence of neuronal or glial tau pathology alone is not specific for CTE, and identification of the pathognomonic CTE lesion remains essential for establishing the diagnosis.

From a gender perspective, CTE has predominantly been reported in men, reflecting both higher male participation in contact sports and potential selection bias in prior studies, which may have underrepresented other sources of RHI such as domestic violence [14, 35]. We applied an unbiased screening approach; although women comprised 55% of our CJD cohort, all three CTE‐positive cases were male with a documented history of contact sports participation. These findings suggest that, in our setting, sport‐related RHI exposure is the primary contributor to CTE, consistent with the historical male predominance in these activities.

This study has several limitations. Due to its retrospective design, detailed clinical and sport exposure information was limited, and contact sports history was not systematically available for CTE‐negative cases. The initial screening was limited to the frontal cortex and hippocampus of a single hemisphere, potentially reducing sensitivity to early pathology, although cases with suggestive features underwent expanded assessment.

Despite these limitations, the study benefits from the systematic evaluation of a large neuropathologically confirmed CJD cohort using current CTE diagnostic criteria and an unbiased screening approach independent of sex or sports exposure. The identification of three high‐grade CTE cases indicates that the methodology effectively detected clinically relevant cases.

## 5 CONCLUSION

This study provides the first systematic assessment of CTE in a neuropathologically confirmed CJD cohort and identified three cases of coexisting high‐stage CTE and CJD (1.4%). These cases also represent the first neuropathologically confirmed diagnoses of CTE reported in Spain. All three occurred in male former contact‐sport athletes with heterogeneous clinical and neuropathological profiles. These findings highlight the importance of systematic documentation of repetitive head impact exposure in neurological and neuropathological evaluations. The coexistence of CTE and CJD raises the possibility of overlapping neurodegenerative mechanisms that warrant further investigation.

## AUTHOR CONTRIBUTIONS

*Conceptualization*: RS‐V, CJN, and LM‐P. *Methodology*: RS‐V, IA, NV, and LM‐P. *Validation*: IA, AS, LB, ACM, CJN, and LM‐P. *Formal analysis, data curation, visualization, and writing – original draft*: LB, IA, and LM‐P. *Investigation*: LB, AS, NV, VS, AG, and LM‐P. *Resources*: VS, AG, RB, CN, and MM‐C. *Writing – review and editing*: all authors. *Supervision*: AI, RS‐V, and LM‐P. *Funding acquisition*: RS‐V.

## FUNDING INFORMATION

This study was supported by the José Baselga Research Fund. Dr José Baselga funds for research in prion diseases.

## CONFLICT OF INTEREST STATEMENT

The authors declare no conflicts of interests.

## ETHICS STATEMENT

The study was approved by the Clinical Research Ethics Committee of Hospital Clínic de Barcelona (HCB/2022/1069) and conducted in accordance with the principles of the Declaration of Helsinki. Brain donation and research procedures were performed according to the regulations of the Biobank‐Hospital Clínic‐IDIBAPS.

## PATIENT CONSENT

Informed consent for brain donation and the use of tissue and associated clinical data for research purposes was obtained according to Biobank‐Hospital Clínic‐IDIBAPS procedures and applicable regulations.

## Data Availability

Anonymized clinical and neuropathological data, as well as biological samples, may be made available upon reasonable request and subject to approval by the Biobank‐Hospital Clínic‐IDIBAPS and in accordance with applicable ethical and legal requirements.

## References

[1] Russell ER , Mackay DF , Lyall D , Stewart K , MacLean JA , Robson J , et al. Neurodegenerative disease risk among former international rugby union players. J Neurol Neurosurg Psychiatry. 2022;93(12):1262–1268. 10.1136/jnnp-2022-329675 PMC9669247 36195436

[2] Gallacher J , Pickering J , Bayer A , Heslop L , Morgan G , Watkins A , et al. Amateur boxing and dementia: cognitive impairment within the 35‐year caerphilly cohort study. Clin J Sport Med. 2022;32(3):329–333. 10.1097/JSM.0000000000000976 35470341

[3] Mackay DF , Russell ER , Stewart K , MacLean JA , Pell JP , Stewart W . Neurodegenerative disease mortality among former professional soccer players. N Engl J Med. 2019;381(19):1801–1808. 10.1056/NEJMoa1908483 PMC8747032 31633894

[4] McKee AC , Cantu RC , Nowinski CJ , Hedley‐Whyte ET , Gavett BE , Budson AE , et al. Chronic traumatic encephalopathy in athletes: progressive tauopathy after repetitive head injury. J Neuropathol Exp Neurol. 2009;68(7):709–735. 10.1097/NEN.0b013e3181a9d503 PMC2945234 19535999

[5] Martland HS . Punch drunk. JAMA. 1928;91(15):1103–1107.

[6] Castellani RJ , Perry G . Dementia pugilistica revisited. J Alzheimer's Dis. 2017;60(4):1209–1221. 10.3233/JAD-170669 PMC5676846 29036831

[7] Bieniek KF , Cairns NJ , Crary JF , Dickson DW , Folkerth RD , Keene CD , et al. The second NINDS/NIBIB consensus meeting to define neuropathological criteria for the diagnosis of chronic traumatic encephalopathy. J Neuropathol Exp Neurol. 2021;80(3):210–219. 10.1093/jnen/nlab001 PMC7899277 33611507

[8] McKee AC , Stein TD , Huber BR , Crary JF , Bieniek K , Dickson D , et al. Chronic traumatic encephalopathy (CTE): criteria for neuropathological diagnosis and relationship to repetitive impacts. Acta Neuropathol. 2023;145(4):371–394. 10.1007/s00401-023-02540-w PMC10020327 36759368

[9] Mez J , Daneshvar DH , Kiernan PT , Abdolmohammadi B , Alvarez VE , Huber BR , et al. Clinicopathological evaluation of chronic traumatic encephalopathy in players of American football. Jama. 2017;318(4):360–370. 10.1001/jama.2017.8334 PMC5807097 28742910

[10] Stewart W , Buckland ME , Abdolmohammadi B , Affleck AJ , Alvarez VE , Gilchrist S , et al. Risk of chronic traumatic encephalopathy in rugby union is associated with length of playing career. Acta Neuropathol. 2023;146:829–832. 10.1007/s00401-023-02644-3 PMC10627955 37872234

[11] Abdoleohammadi B , Tuz‐Zahra F , Uretsky M , Nicks R , Mosaheb S , Labonte J , et al. Duration of ice hockey play and chronic traumatic encephalopathy. JAMA Netw Open. 2024;7(12):e2449106. 10.1001/jamanetworkopen.2024.49106 PMC11618473 39630446

[12] Forrest SL , Kril JJ , Wagner S , Hönigschnabl S , Reiner A , Fischer P , et al. Chronic traumatic encephalopathy (CTE) is absent from a European Community‐based aging cohort while cortical aging‐related tau astrogliopathy (ARTAG) is highly prevalent. J Neuropathol Exp Neurol. 2019;78(5):398–405. 10.1093/jnen/nlz017 30939193

[13] Suter CM , Affleck AJ , Lee M , Davies D , Burns AL , Sy J , et al. Chronic traumatic encephalopathy in a routine neuropathology service in Australia. J Neuropathol Exp Neurol. 2022;81(10):790–795. 10.1093/jnen/nlac071 PMC10622321 35947764

[14] Bieniek KF , Ross OA , Cormier KA , Walton RL , Soto‐Ortolaza A , Johnston AE , et al. Chronic traumatic encephalopathy pathology in a neurodegenerative disorders brain bank. Acta Neuropathol. 2015;130(6):877–889. 10.1007/s00401-015-1502-4 PMC4655127 26518018

[15] Baiardi S , Rossi M , Capellari S , Parchi P . Recent advances in the histo‐molecular pathology of human prion disease. Brain Pathol. 2019;29(2):278–300. 10.1111/bpa.12695 PMC8028685 30588685

[16] Nemani SK , Notari S , Cali I , Alvarez VE , Kofskey D , Cohen M , et al. Co‐occurrence of chronic traumatic encephalopathy and prion disease. Acta Neuropathol Commun. 2018;6(1):140. 10.1186/s40478-018-0643-9 PMC6299534 30563563

[17] McKee AC , Stern RA , Nowinski CJ , Stein TD , Daneshvar DH , Alvarez VE , et al. The spectrum of disease in chronic traumatic encephalopathy. Brain. 2013;136(1):43–64. 10.1093/brain/aws307 PMC3624697 23208308

[18] Ximelis T , Marín‐Moreno A , Espinosa JC , Eraña H , Charco JM , Hernández I , et al. Homozygous R136S mutation in PRNP gene causes inherited early onset prion disease. Alzheimer's Res Ther. 2021;13(1):176. 10.1186/s13195-021-00912-6 PMC8524886 34663460

[19] Parchi P , de Boni L , Saverioni D , Cohen ML , Ferrer I , Gambetti P , et al. Consensus classification of human prion disease histotypes allows reliable identification of molecular subtypes: an inter‐rater study among surveillance centres in Europe and USA. Acta Neuropathol. 2012;124(4):517–529. 10.1007/s00401-012-1002-8 PMC3725314 22744790

[20] Montine TJ , Phelps CH , Beach TG , Bigio EH , Cairns NJ , Dickson DW , et al. National Institute on Aging‐Alzheimer's Association guidelines for the neuropathologic assessment of Alzheimer's disease: a practical approach. Acta Neuropathol. 2012;123(1):1–11. 10.1007/s00401-011-0910-3 PMC3268003 22101365

[21] Braak H , Braak E . Neuropathological stageing of Alzheimer‐related changes. Acta Neuropathol. 1991;82(4):239–259. 10.1007/BF00308809 1759558

[22] Thal DR , Rüb U , Orantes M , Braak H . Phases of A beta‐deposition in the human brain and its relevance for the development of AD. Neurology. 2002;58(12):1791–1800. 10.1212/wnl.58.12.1791 12084879

[23] Mirra SS , Heyman A , McKeel D , Sumi SM , Crain BJ , Brownlee LM , et al. The Consortium to Establish a Registry for Alzheimer's Disease (CERAD). Part II. Standardization of the neuropathologic assessment of Alzheimer's disease. Neurology. 1991;41(4):479–486. 10.1212/wnl.41.4.479 2011243

[24] McKeith IG , Galasko D , Kosaka K , Perry EK , Dickson DW , Hansen LA , et al. Consensus guidelines for the clinical and pathologic diagnosis of dementia with Lewy bodies (DLB): report of the consortium on DLB international workshop. Neurology. 1996;47(5):1113–1124. 10.1212/wnl.47.5.1113 8909416

[25] Nelson PT , Dickson DW , Trojanowski JQ , Jack CR , Boyle PA , Arfanakis K , et al. Limbic‐predominant age‐related TDP‐43 encephalopathy (LATE): consensus working group report. Brain. 2019;142(6):1503–1527. 10.1093/brain/awz099 PMC6536849 31039256

[26] Sáinz Pelayo MDP , Pelayo Vergara R , Albu S , Figueira C . Experiencia con cuatro casos clínicos, ¿puede la encefalopatía traumática crónica estar asociada a un traumatismo craneoencefálico único? [Experience with 4 clinical cases. Traumatic encephalopathy may be associated with a single traumatic brain injury?]. Rehabilitacion. 2022;56(4):383–387. 10.1016/j.rh.2021.04.004 34538654

[27] Postupna N , Rose SE , Gibbons LE , Coleman NM , Hellstern LL , Ritchie KLE , et al. The delayed neuropathological consequences of traumatic brain injury in a community‐based sample. Front Neurol. 2021;16(12):624696. 10.3389/fneur.2021.624696 PMC8008107 33796061

[28] Rodriguez RD , Nowinski C , Suemoto CK , Leite REP , de Lucena Ferretti‐Rebustini RE , Jacob‐Filho W , et al. Clinicopathological study of chronic traumatic encephalopathy pathology in a population‐based cohort. J Neuropathol Exp Neurol. 2026;85(5):425–432. 10.1093/jnen/nlaf150 PMC13127888 41627284

[29] McCann H , Bahar AY , Burkhardt K , Gardner AJ , Halliday GM , Iverson GL , et al. Prevalence of chronic traumatic encephalopathy in the Sydney brain Bank. Brain Commun. 2022;4(4):fcac189. 10.1093/braincomms/fcac189 PMC9356727 35950093

[30] Sarkohi A , Hovenberg A , Hägglund M , Serner A , Peek K . Incidence of head contact events including headers, and potential head injuries at the FIFA futsal world cup Lithuania 2021. Scand J Med Sci Sports. 2025;35(6):e70083. 10.1111/sms.70083 PMC12122905 40442922

[31] Lima Y , Karaçoban L , Serner A , Peek K , Köyağasıoğlu O . Potential head injuries in men's football, futsal and beach soccer: distinct exposure‐adjusted frequency and patterns from a comparative video analysis. Knee Surg Sports Traumatol Arthrosc. 2026;34(3):1107–1117. 10.1002/ksa.70158 PMC12948335 41144707

[32] Kondo K , Kuroiwa Y . A case control study of Creutzfeldt‐Jakob disease: association with physical injuries. Ann Neurol. 1982;11(4):377–381. 10.1002/ana.410110410 7049055

[33] De Pedro Cuesta J , Ruiz Tovar M , Ward H , Calero M , Smith A , Verduras CA , et al. Sensitivity to biases of case‐control studies on medical procedures, particularly surgery and blood transfusion, and risk of Creutzfeldt‐Jakob disease. Neuroepidemiology. 2012;39(1):1–18. 10.1159/000339318 22777385

[34] Kovacs GG , Rahimi J , Strobel T , Lutz MI , Regelsberger G , Streichenberger N , et al. Tau pathology in Creutzfeldt‐Jakob disease revisited. Brain Pathol. 2017;27(3):332–344. 10.1111/bpa.12411 PMC8028936 27377321

[35] Tiemensma M , Byard RW , Vink R , Affleck AJ , Blumbergs P , Buckland ME . Chronic traumatic encephalopathy (CTE) in the context of longstanding intimate partner violence. Acta Neuropathol. 2024;148(1):1. 10.1007/s00401-024-02757-3 PMC11230984 38976081

